# Supplementary material for: Antibiotic prescription practices among prescribers for children under five at public health centers III and IV in Mbarara district
Source: PLoS One. 2020 Dec 28;15(12):e0243868. doi: 10.1371/journal.pone.0243868 (PMC7769467; doi:10.1371/journal.pone.0243868)
Supplement: Attachment — Submitted filename: Response to the reviewers.docx [file pone.0243868.s003.docx]

RESPONSE TO THE REVIEWERS

I would like to appreciate very much all your comments and guidance offered concerning my submission. I would therefore like to respond as follows;

**REVIEWER 1**

1. Typing error corrected as highlighted on page 12 line 12, page 14 line 14
2. Uganda Clinical Guideline 2016 (UCG 2016) and WHO – IMNCI 2012 included in the reference section as advised.
3. Statement about etiology of upper respiratory tract infections and acute watery diarrhea revised to indicate the possibility of bacterial etiology occasionally necessitating antibiotic therapy.
4. Statement concerning Rota viral vaccine campaign to mitigate diarrhea has been added to the text.
5. Abbreviations have been removed from figure 2

**REVIEWER 2**

1. The statement on page 7 of the abstract has been reformulated.
2. I have revised the statement “health worker” so that it accurately describes the prescribers as highlighted.
3. The method section has been made clearer to indicate what we actually did as highlighted in the text.
4. In Uganda, Clinical officer is a title given to health cadres who completed a 3-year course leading to award of diploma in medicine and community health. They are the primary care prescribers in health centre IIIs, IVs, outpatient departments of District and Regional hospitals. Medical officers on the other hand are holders of Bachelor of Medicine and Bachelor of Surgery. In Uganda, Medical officers are employed at the level of health centre IVs and above. They work in both outpatient and inpatient departments.
5. Page 8. In Uganda, there is a policy on the level of care and referral systems. The lowest level being the Village Health Team (VHT), they provide home based care for malaria and pneumonia. VHTs identify and refer patients who may need further care to either Health centre II or III. Health centre II mainly handles vaccination and uncomplicated antenatal care. They refer their patient to health centre IIIs. Health centre IIIs refer to health centre IVs. Both health centre IIIs and IVs have basic laboratory services like microscopy and complete blood count only. Health centre IIIs and IVs can refer to a district hospital. The next level of care in the hierarchy are regional and national referral hospitals, they have capacity to offer specialized care. So, in Uganda from the level IV down to VHTs are referred to as lower level facility since they lack facilities for investigations.
6. There is no any study on antibiotic prescription practices in Mbarara and Uganda in general. This has already been highlighted as a gap that necessitated our study.

The method section has been separated to reflect records and prescribers’ interview as advised.

One limitation has been removed as suggested.

Health centre IIIs and IVs in Uganda have only basic laboratory services, they cannot perform microbiological studies including blood culture.

The conclusion that prescription of antibiotic was irrational in this setting has been revised to reflect components of irrationality assessed in our study.

Reviewer 3

I absolutely agree with you that a lot of literature exist on antibiotic resistance and irrational prescription. We however did not see any published article on antibiotic prescription practices in a rural setting of Uganda. We thought this study was necessary as a baseline for a bigger study with a better design which would inform policy on control and prevention of antibiotic resistance.
