# Supplementary material for: Antibiotic prescription practices among prescribers for children under five at public health centers III and IV in Mbarara district
Source: PLoS One. 2020 Dec 28;15(12):e0243868. doi: 10.1371/journal.pone.0243868 (PMC7769467; doi:10.1371/journal.pone.0243868)
Supplement: Attachment — Submitted filename: Response to the reviewers.docx [file pone.0243868.s004.docx]

**TO: THE ACADEMIC EDITOR PLOS ONE**

**RESPONSE TO THE REVIEWERS**

I would like to appreciate very much all your comments and guidance offered concerning my submission. I would therefore like to respond as follows;

1. **Introduction para 2:**  In Uganda, Clinical officer is a title given to health cadres who completed a 3-year course leading to award of diploma in medicine and community health. They are the primary care prescribers in health centre IIIs, IVs, outpatient departments of District and Regional hospitals. They literally work as assistant medical officers. Medical officers on the other hand are holders of Bachelor of Medicine and Bachelor of Surgery. In Uganda, Medical officers are employed at the level of health centre IVs and above. They work in both outpatient and inpatient departments.
2. **Introduction para 4:** In Uganda, there is a policy on the level of care and referral systems. The lowest level being the Village Health Team (VHT), they provide home based care for malaria and pneumonia. VHTs identify and refer patients who may need further care to either Health centre II (level 2) or III (level 3). Health level 2 mainly handles vaccination and uncomplicated antenatal care. They refer their patients to health centre IIIs (level 3). Health centre IIIs refer to health centre IVs (level 4). Both health centre IIIs (level 3) and IVs (level 4) have basic laboratory services like microscopy and complete blood count only. Health centre IIIs and IVs can refer to a district hospital. The next level of care in the hierarchy are regional and national referral hospitals, they have capacity to offer specialized care. So, in Uganda from the level IV down to VHTs are referred to as lower level facility since they lack facilities for investigations.
3. **Methods:** I have addressed the grammatical errors noted under method section, refer to page 4 and 5 in the text.
4. The double heading ‘Results’ and legends for tables and figures have been revised to avoid repetition.
5. The citations style has been changed to ‘plos’ format
